# Effectiveness of a 40-minute Ophthalmologic Examination Teaching Session on Medical Student Learning

**DOI:** 10.5811/westjem.2015.7.24933

**Published:** 2015-10-20

**Authors:** Wirachin Hoonpongsimanont, Kambria Nguyen, Wu Deng, Dena Nasir, Bharath Chakravarthy, Shahram Lotfipour

**Affiliations:** University of California, Irvine School of Medicine, Emergency Department, Irvine, California

## Abstract

**Introduction:**

Emergency physicians are among the few specialists besides ophthalmologists who commonly perform ophthalmologic examinations using the slit lamp and other instruments. However, most medical schools in the United States do not require an ophthalmology rotation upon completion. Teaching procedural skills to medical students can be challenging due to limited resources and instructor availability. Our study assesses the effectiveness of a 40-minute hands-on teaching session on ophthalmologic examination for medical students using only two instructors and low-cost equipment.

**Methods:**

We performed an interventional study using a convenience sample of subjects. Pre- and post-workshop questionnaires on students’ confidence in performing ophthalmologic examination were administered. We used a paired t-test and Wilcoxon rank test to analyze the data.

**Results:**

Of the 30 participants in the study, the mean age was 25 and the majority were first-year medical students. The students’ confidence in performing every portion of the ophthalmologic exam increased significantly after the teaching session. We found that the average confidence level before the teaching session were below 2 on a 1–5 Likert scale (1 being the least confident). Confidence levels in using the slit lamp had the highest improvement among the skills taught (2.17 95% CI [1.84–2.49]). Students reported the least improvement in their confidence in assessing extraocular movements (0.73, 95% CI [0.30–1.71]) and examining pupillary function (0.73, 95% CI [0.42–1.04]). We observed the biggest difference in median confidence level in the use of the tonometer (4 with a p-value of <0.05).

**Conclusion:**

A 40-minute structured hands-on training session can significantly improve students’ confidence levels in ophthalmologic skills.

## INTRODUCTION

Teaching procedural skills continues to be a major challenge in medical student education. Procedural skill requires knowledge, familiarity with the instrument, and hand-eye coordination. Despite advances in medical education and the availability of various methods for teaching procedural skills, a 2009 survey reported that recent medical school graduates felt a lack of self-confidence in their ability to perform common procedures upon entering residency training.^4^ Conversely, procedural training in medical school is associated with higher self-reported competency with common medical procedures upon entering residency training. Therefore, it is highly desirable to provide medical students with more opportunities to learn hands-on procedural skills.^4^

The ophthalmologic examination, although indispensable in the emergency department (ED), has been less emphasized in medical education.^5^ Most of the medical schools in the U.S. do not require a rotation in ophthalmology or formal ophthalmologic training for medical students.^5^ One study reported that the slit lamp is one of 12 procedures that even emergency medicine (EM) residents felt they were under-prepared to perform.^6^ EM physicians are among the few specialists who commonly perform ophthalmologic examinations using the slit lamp, tonometry, and ophthalmoscope. To remedy the lack of procedural training in ophthalmological procedures, we created a 40-minute structured hands-on skills teaching session for medical students interested in EM. The session required two instructors, low-cost simulated globes, two tonometers and a portable slit lamp. We then examined whether this teaching session showed benefit to a medical student’s confidence in performing ophthalmologic examination.

## METHODS

### Data collection

**Figure 1:**
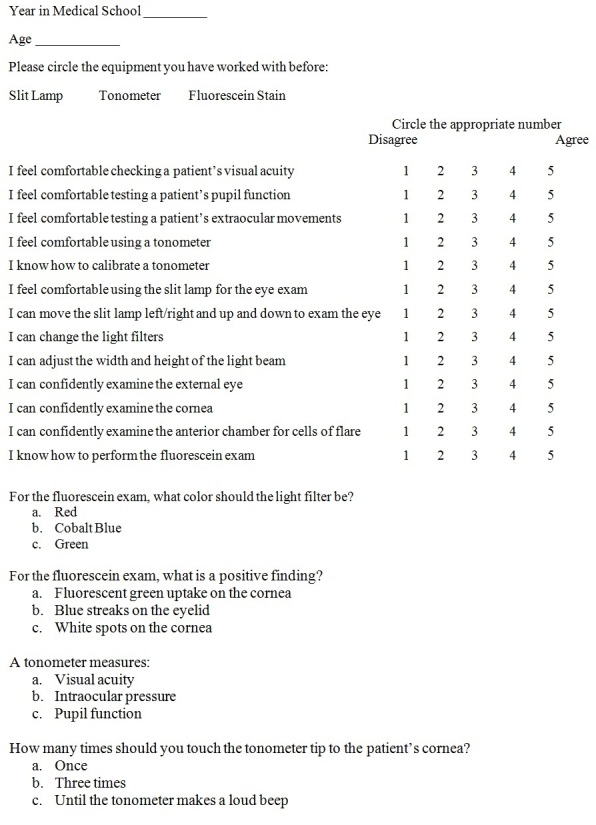
Emergency medicine interest group symposium: ophthalmology workshop questionnaire.

We conducted this cross-sectional study of a convenience sample of 30 medical students at the 2012 Emergency Medicine Interest Group Symposium. The 40-minute teaching session was delivered to 10 medical students at a time. The participants were asked to complete a pre- and post-workshop questionnaire to assess their confidence level with the instruments. We then tabulated the results of the pre- and post-workshop questionnaires in Excel (Microsoft, Redmond, WA) (Figure 1).

### Teaching session

The participants rotated through stations focused on slit lamp, tonometer, and the ophthalmoscope. Students spent 12 minutes at each station and two minutes completing the pre- and post-workshop questionnaires. There were instructors at the slit lamp and tonometry stations. At the ophthalmoscope station, the students were given the opportunity to practice their fundoscopic skills, with an instructor available for questions. We used one portable slit lamp, two tonometers and four ophthalmoscopes.

### Slit Lamp

**Figure 2:**
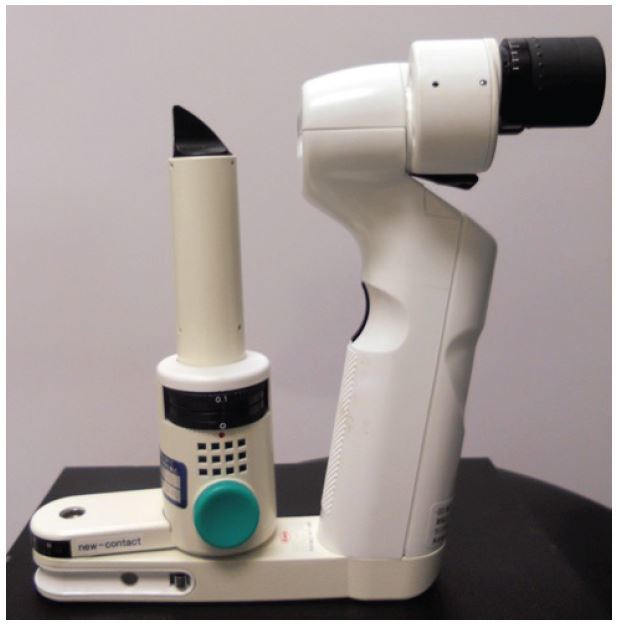
Portable slit lamp.

At the slit lamp station (Keeler PSL, Keeler Ophthalmic Instruments, Broomall, PA), participants used a portable slit lamp to inspect the external eye structures (eyelids, cornea, iris, etc.) and the anterior chamber of their peers’ eyes (Figure 2). After each participant had an opportunity to use the slit lamp, they applied fluorescein stain using paper, and re-examined each other’s eyes. Anesthesia drops were not applied. With only one slit lamp available for use, the remaining students reviewed a slide presentation on other ophthalmologic exam skills and pathology while two students practiced using the slit lamp.

### Tonometry

**Figure 3:**
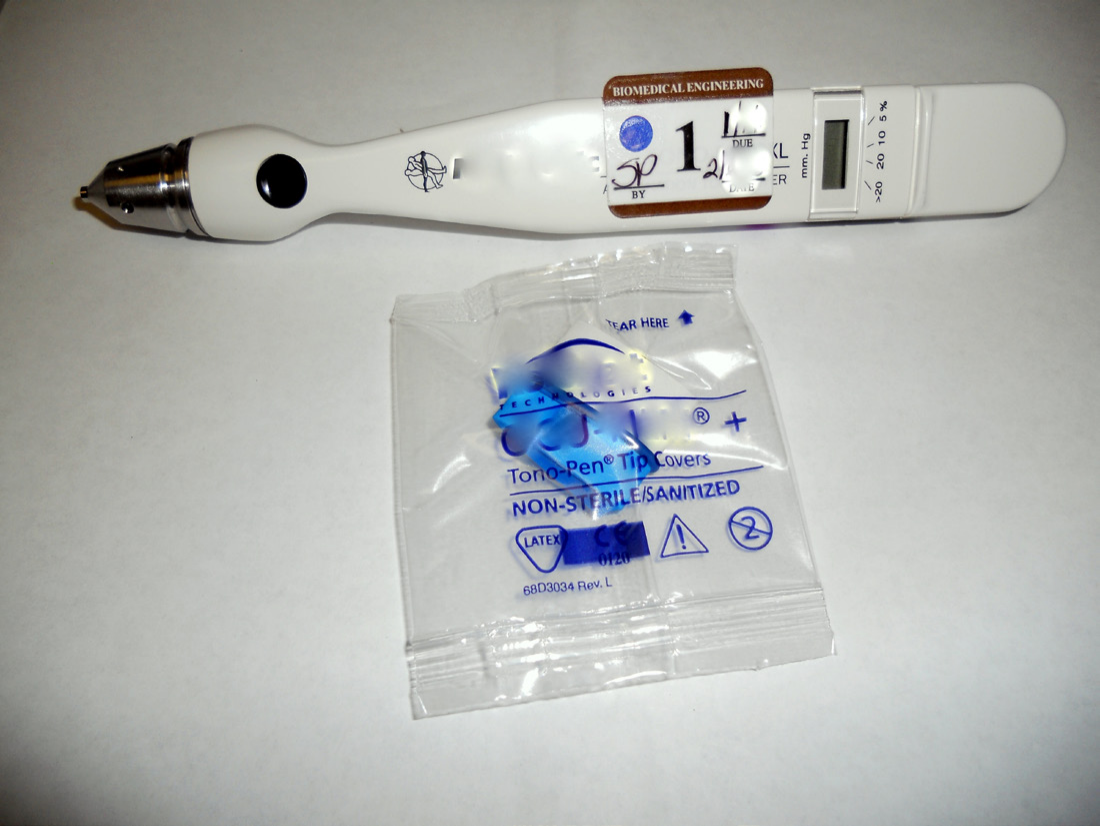
Tonometer.

**Figure 4:**
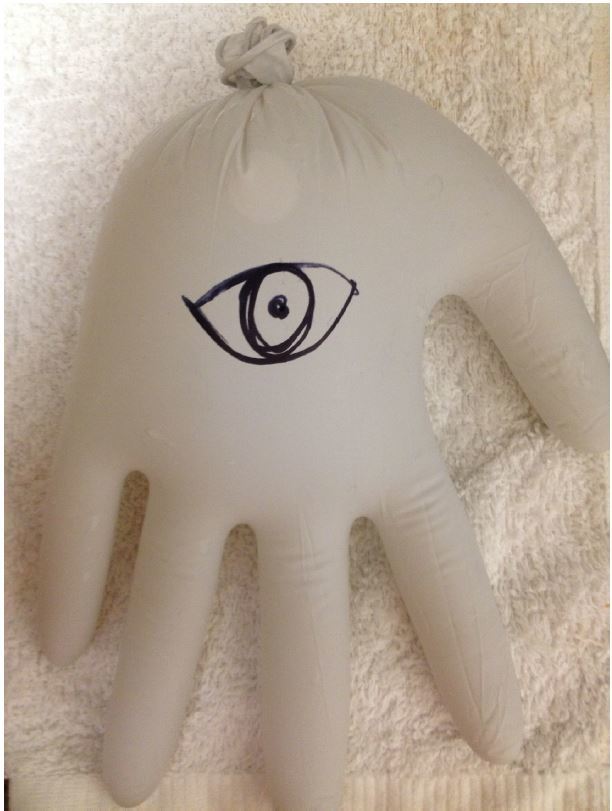
Eye model made of glove filled with water.

At the tonometry station, participants were introduced to the Tonopen (XL, Reichert Technologies, Depew, NY) and how it is used to measure intraocular pressure (Figure 3). The instructor taught the participants to calibrate the Tonopen, apply the protective cover, and assess the intraocular pressure using globe models, made of water-filled gloves (Figure 4).

### Ophthalmoscope and Eye Pathology

**Figure 5:**
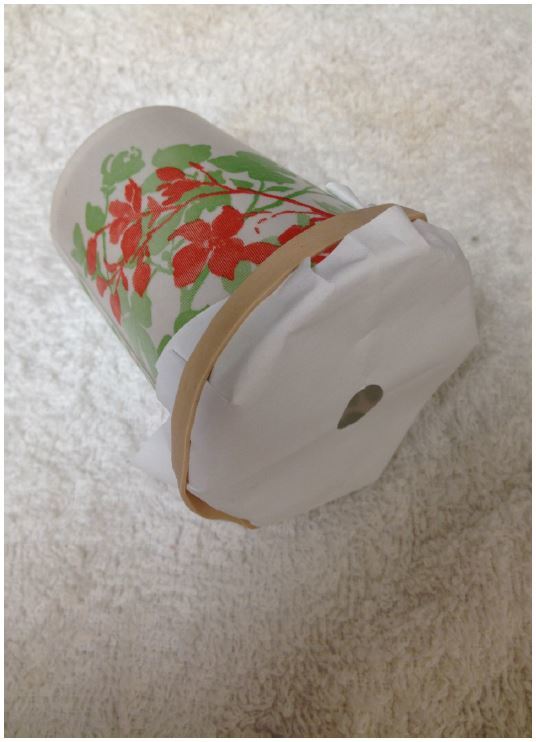
Eye models made from cups with pathology pictures inside. Pathologies included central retinal artery and vein occlusion and retinal detachment.

The ophthalmoscope station had four ophthalmoscopes paired with eye models. These models were made from paper cups with 5 mm opening covers on top (Figure 5). Inside were images of different ophthalmologic pathology. The instructor taught the participants to hold an ophthalmoscope, find the red reflex, how to see the optic disc, and change the light filters. We also had a Panophthalmoscope (Welch Allyn, Skaneateles Fall, NY) for students to practice using.

### Statistical Analysis

We hypothesized that the 40-minute teaching session would improve medical students’ confidence in their ophthalmologic examination skills. The difference in confidence levels between pre- and post-workshop questionnaires was measured on each of the ophthalmologic examination skills listed below:

1. Checking visual acuity
2. Testing pupillary function
3. Testing extraocular movements
4. Using a Tonopen
5. Calibrating a Tonopen
6. Using a slit lamp
7. Examining the external eye
8. Examining the cornea
9. Examining the anterior chamber of the eye
10. Performing a fluorescein examination

We used the paired t-test for dependent variables with normal distributions and the Wilcoxon-rank test for non-parametric dependent variables.

## RESULTS

Of the 30 students, the mean age was 25. Almost two-thirds (19) of the participants were male. The majority of the subjects (19) were first-year medical students with no prior experience performing an ophthalmologic examination (Table 1).

We found that the average confidence level before the teaching session were below 2 on a 1–4 Likert scale (1 being the least confident). Confidence levels in using the slit lamp had the highest improvement (2.17 95% CI [1.84–2.49]). Students reported the least improvement in their confidence in testing extraocular movements (0.73, 95% CI [0.30–1.71]) and pupillary function (0.73, 95% CI [0.42–1.04]) (Table 2). Table 2 shows the mean confidence level for each ophthalmologic examination that had normal distribution. The differences in confidence level between before and after teaching sessions were statistically significant.

**Figure 6:**
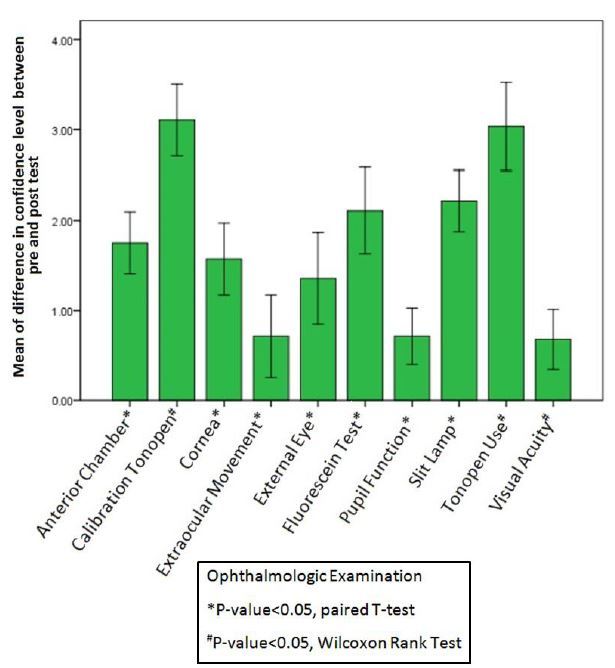
Difference in confidence level before and after an ophthalmology teaching session.

Table 3 shows the median of confidence level for each ophthalmologic examination that had non-normal distribution. We observed the biggest difference in the median of confidence levels for tonometer use (4, p-value <0.05), which was statistically significant when compared before and after the teaching session. Overall the improvement in confidence levels was statistically significant across all portions of the ophthalmologic examination after completing the 40-minute teaching session (Figure 6).

## DISCUSSION

Multiple methods have been explored to improve the efficiency of teaching procedure skills. The methods include computer-aided programs, simulations, and cadaver labs, which show convincing evidence of success, although they can be cost prohibitive in many instances.^1^,^2^ Practicing procedures on real patients has been debated due to safety issues and patient dissatisfaction.^8^,^10^ Using cadavers or animal models is expensive and limited by availability.^9^ Computer interactive courses and virtual simulations have been considered by many educators as being equal to clinical skills workshops. A randomized study of both nursing students and medical students suggested that traditional hands-on training was superior to an interactive, virtual-reality computer intravenous catheter simulation.^3^

The “see one, do one, teach one” concept emphasizes the necessity of learning procedures by doing instead of observation.^7^ Educators realize that a competency gap exists between the “see one, do one” model. Additional hands-on sessions under supervision are necessary to address the gap.^8^,^9^ We designed a brief interactive workshop using low-cost equipment to improve students’ familiarity with equipment and their hand-eye coordination skills.

In our workshop, the medical students practiced on each other after they became comfortable with the skill sets. This strategy provided hands-on experience on human subjects utilizing limited resources. It served as a bridge between cognitive understanding and actual manual skills. In addition, the workshop did not involve a formal lecture. We demonstrated that teaching procedural skills does not require in-depth medical knowledge to improve a practitioner’s confidence.

The success of our workshop might also be attributed to the well-focused curriculum. We realized it was not possible to cover all the ophthalmologic examinations in depth in 40 minutes. Because our target audience was medical students interested in EM rather than ophthalmology, we selected three of the most commonly used ophthalmologic instruments: the slit lamp, tonometer, and ophthalmoscope. While the results of our study indicated that our 40-minute workshop resulted in significant improvement of confidence levels for the three skills, they also suggested only short-term improvement of confidence level in ophthalmologic examination. Future studies should implement a direct observation session to assess performance and retention of procedural skills in addition to self-reported confidence. However, the retention of procedural skills is unpredictable and different from the retention of medical knowledge. For example, a study on a short-course cardiopulmonary resuscitation training reported significantly lower skill retention after five months, whereas a study on simulation-based mastery learning of central venous line insertions reported one-year retention of acquired skills.^11^

Our study demonstrates that short but structured procedural skill workshops can increase students’ confidence levels before they enter clinical years. We observed significant improvement in procedural skills that use equipment. These procedures include slit lamp examination, fluorescein staining and tonometry. The results supported the idea that familiarity with equipment is an essential part of learning procedural skills. Therefore, educators should provide trainees ample access to procedural equipment. The opportunity to operate the equipment prior to performing procedures in live subjects may enhance a student’s confidence significantly. Both EM residency and medical student educators could easily implement this workshop to increase hands-on experience and confidence levels when performing ophthalmologic examinations.

## LIMITATIONS

Our study had several limitations. While we showed significant findings despite the small sample size, there are no currently validated tools designed specifically to assess procedural competency.^9^ Therefore, we chose to use a pre- and post-test study design. Our outcome measures were temporally related to our intervention. We did not test the subjects’ confidence level with the skills at a later date. Neither did we assess the educator’s evaluation of the subjects performing these skills. Future studies should also evaluate the students’ procedural competency with real patients. For example, diagnosis of an ophthalmologic pathology in a globe model is greatly different from diagnosing a patient with a moving globe and small pupils.

## CONCLUSION

Teaching procedural skills to medical students is a challenge in medical education. We created a 40-minute teaching session consisting of three stations focusing on tonometry, slit lamp, and fundoscopy. The session used two instructors and low-cost resources. We then used a short pre and post questionnaire to evaluate students’ confidence levels. Our study demonstrated that this hands-on workshop significantly improved students’ confidence in ophthalmologic examination, especially in using slit lamp and tonometry.

## Figures and Tables

**Table 1:** Medical student demographic data in study examining student confidence in performing ophthalmologic exams.

Characteristic	Number (%)
Age (yr) (n=30)	Mean 25.03 (SD 2.86)
Medical school year (n=30)	
Year 1	19 (63.33)
Year 2	4 (13.33)
Year 3	2 (6.77)
Year 4	2 (6.77)
Other	3 (10.00)
Gender (n=30)	
Male	19 (63.33)
Female	11 (36.77)
Past experience with ophthalmology rotation (n=30)	0 (0.00)
Fluorescein exam (n=21)	2 (6.77)
Slit lamp exam (n=21)	4 (13.33)
Tonopen (n=21)	1 (3.33)

**Table 2:** Comparison of mean confidence levels in individual ophthalmologic examinations using a student t-test.

Ophthalmologic examination	Mean of pre-test confidence level (SD)	Mean of post-test confidence level (SD)	Mean: difference confidence level (95% CI)	P-value
Anterior chamber	1.17 (0.46)	2.90 (0.92)	1.73 (1.41–2.06)	<0.05
Cornea	1.79 (1.01)	3.38 (0.94)	1.59 (1.20–1.97)	<0.05
EOM	2.43 (1.65)	3.17 (1.46)	0.73 (0.30–1.71)	<0.05
External eye	2.17 (1.32)	3.50 (1.14)	1.33 (0.85–1.81)	<0.05
Fluorescein test	1.23 (0.68)	3.27 (1.31)	2.03 (1.57–2.50)	<0.05
Pupil function	2.57 (1.68)	3.30 (1.44)	0.73 (0.42–1.04)	<0.05
Slit lamp	1.27 (0.64)	3.43 (0.82)	2.17 (1.84–2.49)	<0.05

*EOM*, extraocular muscles

**Table 3:** Comparison of median of confidence levels in individual ophthalmologic examination using the Wilcoxin rank sum test.

Ophthalmologic examination	Median of pre-test confidence level (IQR)	Median of post-test confidence level (IQR)	P-value
Calibration of tonometer	1.00 (0.00)	4.00 (1.25)	<0.05
Tonometer use	1.00 (0.00)	5.00 (2.00)	<0.05
Visual acuity	3.00 (3.25)	4.00 (1.25)	<0.05
